# The anti-CD74 humanized monoclonal antibody, milatuzumab, which targets the invariant chain of MHC II complexes, alters B-cell proliferation, migration, and adhesion molecule expression

**DOI:** 10.1186/ar3767

**Published:** 2012-03-09

**Authors:** Daniela Frölich, Daniela Blaβfeld, Karin Reiter, Claudia Giesecke, Capucine Daridon, Henrik E Mei, Gerd R Burmester, David M Goldenberg, Abdulagabar Salama, Thomas Dörner

**Affiliations:** 1 CC12 Department of Medicine, Rheumatology and Clinical Immunology, Charité - University Medicine Berlin, Chariteplatz 1, Berlin 10117, Germany; 2 Deutsches Rheumaforschungszentrum (DRFZ), Charité - University Medicine Berlin, Chariteplatz 1, Berlin 10117, Germany; 3 Immunomedics, Inc., and Center for Molecular Medicine and Immunology, 300 The American Road, Morris Plains, NJ 07950, USA; 4 CC14 Institute of Transfusion Medicine, Charité - University Medicine Berlin, Chariteplatz 1, Berlin 10117, Germany

## Abstract

**Introduction:**

Targeting CD74 as the invariant chain of major histocompatibility complexes (MHC) became possible by the availability of a specific humanized monoclonal antibody, milatuzumab, which is under investigation in patients with hematological neoplasms. CD74 has been reported to regulate chemo-attractant migration of macrophages and dendritic cells, while the role of CD74 on peripheral naïve and memory B cells also expressing CD74 remains unknown. Therefore, the current study addressed the influence of milatuzumab on B-cell proliferation, chemo-attractant migration, and adhesion molecule expression.

**Methods:**

Surface expression of CD74 on CD27^- ^naïve and CD27^+ ^memory B cells as well as other peripheral blood mononuclear cells (PBMCs) obtained from normals, including the co-expression of CD44, CXCR4, and the adhesion molecules CD62L, β7-integrin, β1-integrin and CD9 were studied after binding of milatuzumab using multicolor flow cytometry. The influence of the antibody on B-cell proliferation and migration was analyzed *in vitro *in detail.

**Results:**

In addition to monocytes, milatuzumab also specifically bound to human peripheral B cells, with a higher intensity on CD27^+ ^memory versus CD27^- ^naïve B cells. The antibody reduced B-cell proliferation significantly but moderately, induced enhanced spontaneous and CXCL12-dependent migration together with changes in the expression of adhesion molecules, CD44, β7-integrin and CD62L, mainly of CD27^- ^naïve B cells. This was independent of macrophage migration-inhibitory factor as a ligand of CD74/CD44 complexes.

**Conclusions:**

Milatuzumab leads to modestly reduced proliferation, alterations in migration, and adhesion molecule expression preferentially of CD27^- ^naïve B cells. It thus may be a candidate antibody for the autoimmune disease therapy by modifying B cell functions.

## Introduction

Milatuzumab is a humanized monoclonal antibody (also called hLL1 or IMMU-115) targeting a cell surface-expressed epitope of the molecule CD74 which is expressed on monocytes, macrophages, and B cells but not T cells [1]. Currently, milatuzumab is a candidate immunotherapeutic antibody being studied in multiple myeloma, non-Hodgkin lymphoma, and chronic lymphocytic leukemia (CLL) [1]. B cells of these hematological tumors express the target molecule, CD74, at high levels and internalize it rapidly after milatuzumab binding [2,3]. Consequently, growth inhibition and induction of apoptosis in B-cell lines in the presence of a second cross-linking antibody (for example, Fcγ-specific F(ab)_2 _fragments to mimic the role of effector cells or cross-linking molecules present *in vivo*) have been reported [4]. Therefore, milatuzumab seems to block CD74 signaling pathways and act as an antagonist. In preclinical models, treatment of cynomolgus monkeys with milatuzumab led to a decrease of peripheral blood mononuclear cells (PBMCs) but not to systemic toxicity or enhanced mortality [1].

The target molecule of milatuzumab, CD74, is a transmembrane glycoprotein that associates with the major histocompatibility complex (MHC) class II α and β chains and is also known as MHC class II invariant chain (Ii). In this context, CD74 functions as a chaperone molecule and is implicated in antigen presentation [5,6]. Moreover, CD74 is involved in several signaling pathways of B-cell survival. In particular, binding of macrophage migration inhibitory factor (MIF), a chemokine produced by a variety of cell types, including monocytes and lymphocytes, to a receptor complex formed by CD74 and CD44 initiates a signaling cascade in B cells which involves spleen tyrosine kinase (Syk), phosphatidylinositol 3-kinase (PI3K), and Akt, leading to nuclear factor-kappa-B (NF-κB) activation, transcription of anti-apoptotic genes, and (finally) cell survival and proliferation [7-10]. Another CD74-dependent anti-apoptotic pathway promotes B-CLL cell growth and survival by activating p65 (a member of the NF-κB family), upregulating expression of the transactivation isoforms of p63 (TAp63), and inducing Bcl-2 expression and interleukin-8 (IL-8) secretion [2,11,12]. Furthermore, CD74 has been shown to be involved in B-cell maturation by activating a TAF_II_105-NF-κB-dependent transcription program [13,14]. A recently detected complex formed by CD74 and CXCR4 acts as an alternative functional MIF receptor, resulting in phosphorylation of Akt in Jurkat cells [15]. Since CXCR4 is known as the receptor involved in the migration of B cells toward CXCL12 [16,17], it is not clear to which extent this function can be influenced by the anti-CD74 antibody, milatuzumab.

Previous studies addressing the function of milatuzumab are based on B-cell lines or B cells that are from patients with CLL and that express CD74 at high levels or mouse splenocytes and cell lines [1,4]. In contrast, there is a lack of knowledge about the effects of milatuzumab on B cells from healthy individuals or from patients with non-malignant diseases. Therefore, the present study analyzed the surface expression of CD74 and the related molecules, CD44 and CXCR4, as well as the functional impact of milatuzumab on B cells from healthy donors, including B-cell proliferation and migration toward CXCL12. Furthermore, the influence of milatuzumab on the co-expression of β1-integrin, CD9, β7-integrin, and CD62L involved in cell adhesion was investigated.

## Materials and methods

### Subjects and cell preparation

Peripheral blood from 32 healthy Caucasian individuals (24 females and 8 males) with a mean age of 34 years (range of 19 to 66 years) was collected after informed consent was obtained and approval was granted by the ethics committee of the Charité University Medicine Berlin. PBMCs from some individuals were used for different experiments on different days. PBMCs were prepared by density gradient centrifugation by using Ficoll-Paque Plus (GE Healthcare Bio-Sciences AB, Uppsala, Sweden), as reported previously [18]. Washing steps were performed depending on further processing with cold phosphate-buffered saline (PBS)/0.2% bovine serum albumin (BSA) (for direct cell staining), PBS (for proliferation experiments), or pre-warmed RPMI 1640 medium supplemented with 0.5% BSA (assay medium for transwell migration assays and incubation experiments). Cells were stained for flow cytometric analyses immediately or after *in vitro *experiments.

### Flow cytometry

PBMCs were incubated at 4°C for 15 minutes with the following fluorochrome-labeled anti-human monoclonal antibodies in PBS/0.2% BSA: anti-CD19-phycoerythrin-Cy7 (PE-Cy7, clone SJ25C1; BD Biosciences, San Jose, CA, USA), anti-CD3-Pacific Blue (PacB, clone UCHT1; BD Biosciences), anti-CD14-PacB (clone M5E2; BD Biosciences), anti-CD27-Cy5 (clone 2E4, kind gift from René van Lier, Academic Medical Center, University of Amsterdam, The Netherlands), anti-CD74-FITC (clone M-B741; BD Biosciences), milatuzumab-PE (hLL1; Immunomedics, Inc., Morris Plains, NJ, USA, labeled in-house at the Deutsches Rheumaforschungszentrum), anti-CD44-FITC (clone L178; BD Biosciences), anti-CXCR4-PE (clone 12G5; BD Biosciences), anti-β1-integrin-PE (clone MAR4; BD Biosciences), anti-CD62L-FITC (clone 145/15; Miltenyi Biotec GmbH, Bergisch Gladbach, Germany), and anti-CD9-FITC (clone MM2/57; Chemicon, Schwalbach, Germany). Rat anti-mouse anti-β7-integrin-PE (clone FIB504; BD Biosciences), which cross-reacts with human β7-integrin, was used to detect human β7-integrin, as described before [19-21]. Specificity of staining was confirmed by corresponding isotype controls or blocking by using unconjugated milatuzumab (20-fold concentration). Pre-incubation of PBMCs with unconjugated milatuzumab for 15 minutes on ice did not have an impact on the staining and detection with anti-CD62L-FITC, anti-CD44-FITC, and anti-β7-integrin-PE, so potential sterical interference could be largely excluded.

DAPI (4,6 diamidino-2-phenylindole) (Molecular Probes, now part of Invitrogen Corporation, Carlsbad, CA, USA) was added immediately before cytometric analysis to determine or exclude dead cells. Flow cytometric data were acquired by using a FACSCanto II analyzer (BD Biosciences) and analyzed by using FCS Express 3.0 (De Novo Software, Thornhill, ON, Canada).

CD19^-^/CD3^+^/SSC^low ^T cells, CD19^-^/CD14^+^/SSC^high ^monocytes, and CD19^+^/CD3^-^/CD14^- ^B cells were analyzed for CD74 expression and milatuzumab binding. CD19^+^/CD3^-^/CD14^-^/CD27^- ^naïve B cells and CD19^+^/CD3^-^/CD14^-^/CD27^+ ^memory B cells were also analyzed for expression of CD44 and CXCR4.

### Proliferation experiments and determination of macrophage migration inhibitory factor concentrations

For proliferation experiments, PBMCs were labeled with carboxyfluorescein succinimidyl ester (CFSE) in accordance with the instructions of the manufacturer (Molecular Probes/Invitrogen Corporation). In 96-well plates (U-bottom; Greiner Bio-One GmbH, Frickenhausen, Germany), approximately 1 × 10^6 ^PBMCs in 250 μL of pre-warmed RPMI 1640 medium, supplemented with fetal calf serum (10%; Invitrogen, Darmstadt, Germany) and penicillin/streptomycin (1 U/mL; Sigma-Aldrich Chemie GmbH, Munich, Germany) with or without unconjugated milatuzumab (end concentration of 10 μg/mL; Immunomedics, Inc.) or as control with intravenous immunoglobulin (IVIG) (end concentration of 10 μg/mL; Octapharma AG, Lachen, Switzerland) were stimulated with IL-2 (end concentration of 20 ng/mL; R&D Systems, Minneapolis, MN, USA), IL-10 (end concentration of 20 ng/mL; R&D Systems), and CpG 2006 (end concentration of 0.3 nmol/mL; TIB Molbiol GmbH, Berlin, Germany) in the presence of cross-linking F(ab)_2 _(end concentration of 2 μg/mL; Dianova, Hamburg, Germany) for 7 days at 37°C in 5% CO_2_. Thereafter, the plates were centrifuged and the supernatants were collected and stored at -20°C for later determination of MIF concentrations. The cells were washed with PBS/0.2% BSA and stained for subsequent cytometric analyses. CD19^+^/CD3 ^-^/CD14^- ^B cells were analyzed for their proliferation by using CFSE labeling.

MIF concentrations in the supernatants, naturally produced by the cultured PBMCs, were determined with a quantitative sandwich enzyme immunoassay in accordance with the instructions of the manufacturer (R&D Systems). Optical density was measured by using an Anthos HTII microplate reader (450 and 578 nm; Anthos Labtec Instruments GmbH, Wals, Austria). MIF concentrations were calculated by using an MIF standard solution.

### Transwell migration assays

Migration assays with PBMCs were performed by using 24-well plates with transwell inserts (diameter of 6.5 mm and pore size of 5 μm; Corning Life Sciences, Acton, MA, USA) coated with fibronectin, as described previously [22]. The lower chamber contained 600 μL of assay medium supplemented with CXCL12 (end concentration of 50 nM; R&D Systems), and baseline migration was defined as spontaneous migration in the absence of chemokine. The upper chamber contained 1 to 2 × 10^6 ^PBMCs in 100 μL of assay medium with or without unconjugated milatuzumab (hLL1, end concentration 10 μg/mL), and human IgG1 (end concentration of 10 μg/mL; Sigma-Aldrich Chemie GmbH) or IVIG (end concentration of 10 μg/mL) served as a control. After 90 minutes of incubation at 37°C in a humid 5% CO_2 _atmosphere, non-migrated cells (upper chamber) and migrated cells (lower chamber) were collected, washed with PBS/0.2% BSA, and stained for cytometric analyses. CD19^+^/CD3^-^/CD14^-^/CD27^-/+ ^B cells were enumerated, and frequencies of migrated cells were calculated with this formula: [migrated cells/(migrated + non-migrated cells)] × 100.

### Incubation experiments

For incubation experiments, 24-well plates with flat-bottom wells (Greiner Bio-One GmbH) containing an average of 2 × 10^6 ^PBMCs in 1 mL of assay medium with or without unconjugated milatuzumab (end concentration of 10 μg/mL) or IgG1 (end concentration of 10 μg/mL) or IVIG (end concentration of 10 μg/mL) as controls were incubated for 90 minutes at 37°C in 5% CO_2_. Thereafter, cells were collected, washed with PBS/0.2% BSA, and stained for cytometric analyses. CD19^+^/CD3^-^/CD14 ^-^/CD27^-/+ ^B cells were analyzed for the expression of CD44, CXCR4, β1-integrin, CD9, β7-integrin, and CD62L.

### Statistical analyses

**Figure 1:**
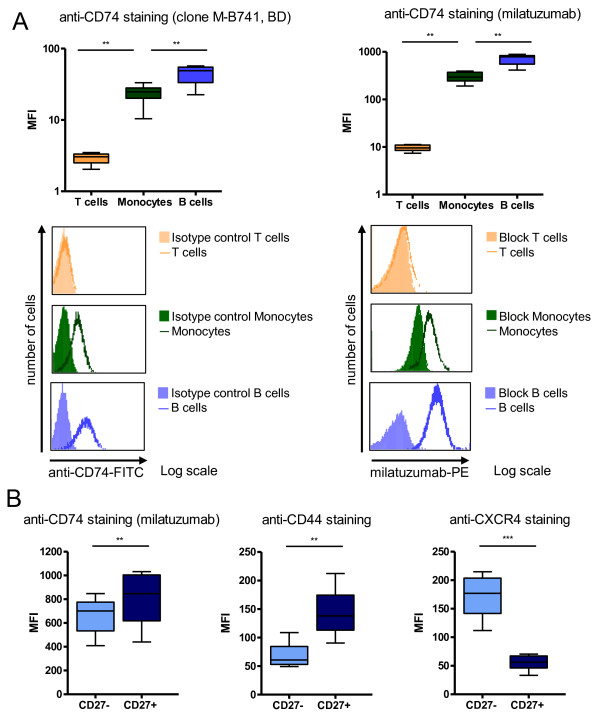
**Surface expression of CD74, CD44, and CXCR4 on T cells, monocytes, and B cells**. **(A) **Detection of CD74 with a commercially available FITC-labeled anti-CD74 antibody (*n *= 8) and PE-labeled milatuzumab (*n *= 9) on T cells, monocytes, and B cells. For each staining, representative histograms, including an isotype control or blocking experiment, are shown. Competitive blocking experiments were performed by using unlabeled milatuzumab (20-fold concentration). Significant differences were observed between the CD74 expression levels of T cells, monocytes, and B cells (Wilcoxon test), and specificity of the staining was confirmed. **(B) **Detection of CD74 (*n *= 10), CD44 (*n *= 8), and CXCR4 (*n *= 12) on CD27^- ^naïve and CD27^+ ^memory B cells. These surface molecules showed a distinct expression profile between these B-cell subpopulations (Wilcoxon test). **P ≤ 0.01; ***P ≤ 0.001. BD, BD Biosciences; FITC, fluorescein isothiocyanate; MFI, (geometric) mean fluorescence intensity; PE, phycoerythrin.

**Figure 2:**
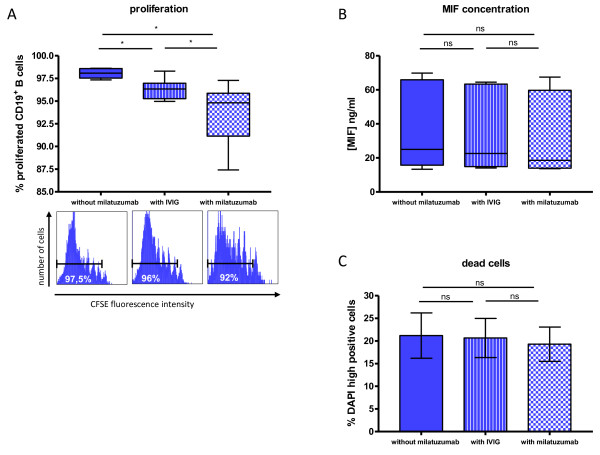
**Effects on proliferation of CD19^+ ^B cells and macrophage migration inhibitory factor (MIF) concentration *in vitro *by milatuzumab**. **(A) **Frequency of proliferated CD19^+^/CD3 ^-^/CD14^- ^B cells according to their carboxyfluorescein succinimidyl ester (CFSE) fluorescence intensity. CFSE-labeled peripheral blood mononuclear cells were cultured for 7 days with or without milatuzumab or intravenous immunoglobulin (IVIG) at 37°C in 5% CO_2 _and simultaneously stimulated with IL-2, IL-10, F(ab)_2_, and CpG (*n *= 6). Addition of milatuzumab as well as IVIG resulted in a modest, but significant, inhibition of the proliferation (Wilcoxon test). For each condition, a representative histogram is shown. **(B) **The concentration of the chemokine MIF as a potential ligand of CD74 was tested in cell culture supernatants (*n *= 7), as described above, and showed no significant differences between the conditions (Wilcoxon test). **(C) **Proportion of dead CD19^+ ^B cells, identified as high positive staining with DAPI (*n *= 3). There was no substantial influence observed by either IVIG or milatuzumab (Wilcoxon test). *P ≤ 0.05. CpG, cytosine-phosphatidyl-guanosine; DAPI, 4,6 diamidino-2-phenylindole; F(ab)_2_, protein of two antigen-binding fragments; IL, interleukin; ns, not significant.

**Figure 3:**
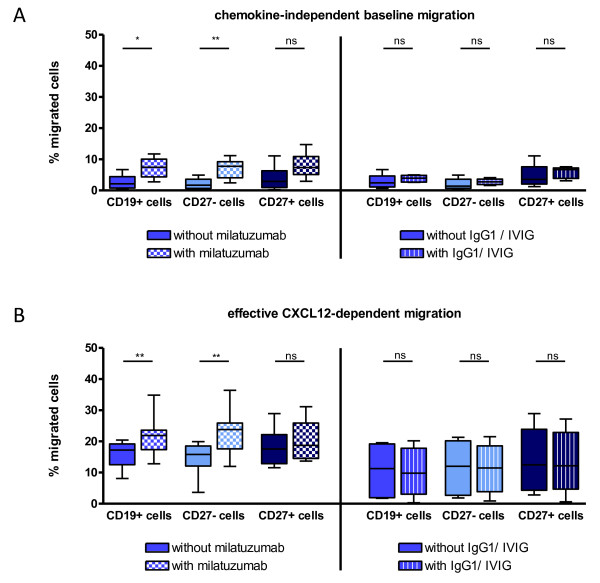
**Influence of milatuzumab on the migration characteristics of CD19^+ ^B cells and subsets *in vitro***. **(A) **Spontaneous baseline migration of B cells in a transwell migration assay. Peripheral blood mononuclear cells were incubated for 90 minutes at 37°C in 5% CO_2 _with or without milatuzumab (*n *= 8) or IgG1/IVIG (*n *= 6) and allowed to migrate without any chemokine added. Percentages of migrated cells are shown. Migration was significantly enhanced (Wilcoxon test) by milatuzumab in CD19^+ ^B cells, especially in CD27^- ^naïve B cells. **(B) **Effective CXCL12-dependent net migration determined for each individual by subtraction of the baseline migration - see (A) - from the total migration toward CXCL12 (not shown). A significant enhancement of the migration of CD19^+ ^B cells, especially of CD27^- ^B cells by milatuzumab, was identified (Wilcoxon test). In control experiments, IgG1/IVIG had no influence on migration. *P ≤ 0.05; **P ≤ 0.01. CXCL, CXC motif ligand; IVIG, intravenous immunoglobulin; ns, not significant.

**Figure 4:**
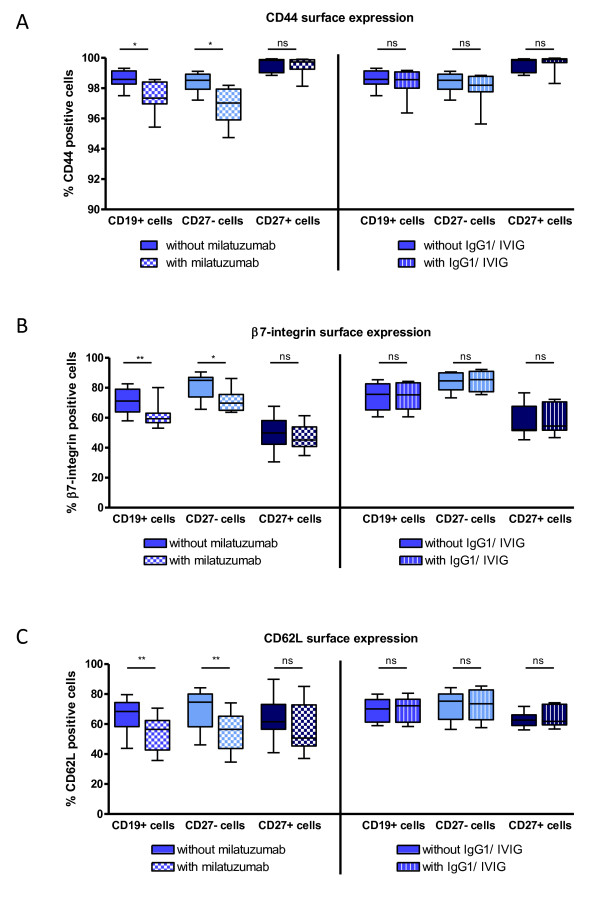
**Surface expression of CD44, β7-integrin, and CD62L on CD19^+ ^B cells was preferentially reduced by milatuzumab on CD27^- ^naïve B cells**. **(A) **Surface expression of CD44 after incubation of peripheral blood mononuclear cells for 90 minutes at 37°C in 5% CO_2 _with or without milatuzumab (*n *= 7) or IgG1/IVIG (*n *= 7). Milatuzumab led to a significant reduction of the expression of CD44 on CD19^+ ^B cells, especially on CD27^- ^naïve B cells (Wilcoxon test). **(B) **Expression of β7-integrin was significantly reduced (Wilcoxon test) on CD19^+ ^B cells, mainly confined to CD27^- ^naïve B cells after milatuzumab incubation (*n *= 8), but not influenced by IgG1/IVIG (*n *= 7). **(C) **Milatuzumab (*n *= 9) significantly reduced the expression of CD62L (Wilcoxon test) on CD19^+ ^B cells, especially on CD27^- ^naïve B cells, whereas IgG1/IVIG (*n *= 8) did not modify this expression. The expression of the surface molecules CD44, β7-integrin, and CD62L on CD27^+ ^memory B cells was not influenced by milatuzumab. *P ≤ 0.05; **P ≤ 0.01. IVIG, intravenous immunoglobulin; ns, not significant.

GraphPad Prism version 4 for Windows (GraphPad Software, San Diego, CA, USA) was used for statistical analyses. Wilcoxon tests were performed to compare different experimental conditions or cell populations within one individual (paired data). *P *values of not more than 0.05 (which were considered significantly different), of not more than 0.01, and of not more than 0.001 are indicated by a single, double, and triple asterisk, respectively, in Figures 1, 2, 3 and 4.

## Results

### Distinct surface expression of CD74, CD44, and CXCR4 on CD19^+^/CD27^- ^naïve versus CD19^+^/CD27^+ ^memory B cells

Initially, direct labeling of PBMCs was performed to determine the surface expression of CD74 on B cells, T cells, and monocytes (Figure 1A). The surface expression of CD44 and CXCR4, molecules reported to be associated with CD74 expression, was also analyzed on human CD27^- ^naïve and CD27^+ ^memory B-cell subpopulations (Figure 1B).

With a commercial anti-CD74 antibody used as a control, T cells did not express CD74 - geometric mean fluorescence intensity (MFI) of 2.9 ± 0.5, mean ± standard deviation (SD) - whereas monocytes (MFI of 23.7 ± 7.1) and B cells (MFI of 44.3 ± 13.6) expressed this molecule at significantly higher levels. The specificity of CD74 staining was confirmed by isotype controls, and the same binding characteristics were confirmed by using fluorescence-labeled milatuzumab. When the MFI was analyzed, milatuzumab did not bind to T cells (MFI of 9.6 ± 1.3) but showed substantial binding to monocytes (MFI of 304 ± 70) and B cells (MFI of 704 ± 175). To confirm the specificity of this binding, unconjugated milatuzumab was added to the staining. Both anti-CD74 antibodies competed for the same epitopes, so the more concentrated unconjugated milatuzumab prevented binding of conjugated milatuzumab. This blocking was seen only partially on monocytes but completely on B cells analyzed in all further experiments. Therefore, milatuzumab binding to B cells appeared to be highly specific among PBMCs.

Almost all peripheral blood B cells expressed CD74 and were recognized by milatuzumab. In detail, milatuzumab binding was detected on 98.3% ± 1.2% of CD27^- ^naïve and 97.0% ± 2.4% of CD27^+ ^memory B cells (data not shown), but for each individual analyzed, the MFI of CD74 binding on CD27^+ ^memory B cells was higher than those on CD27^- ^naïve B cells (by an average of 1.3-fold; *P *= 0.002) (Figure 1B). Surface expression of the co-receptor of CD74, CD44, was identified on almost all B cells (96.7% ± 2.0% of CD27^- ^naïve and 99.3% ± 0.9% of CD27^+ ^memory B cells; data not shown), whereas the density of CD44 expression (MFI) on CD27^+ ^B cells was significantly higher than that on CD27^- ^B cells (by an average of twofold; *P *= 0.0078). In contrast, only 82.2% ± 6.6% of CD27^+ ^B cells expressed CXCR4, whereas 98.0% ± 1.1% of CD27^- ^B cells expressed this molecule (data not shown). Accordingly, the MFI of CXCR4 on CD27^- ^naïve B cells was significantly higher (by an average of 3.1-fold; *P *= 0.0005) than the MFI of CD27^+ ^memory B cells. It should be noted that no correlation was observed between the individual expression levels of CD74, CXCR4, and CD44.

### Milatuzumab moderately inhibits *in vitro *proliferation of CD19^+ ^B cells

CD74, as the invariant chain of the MHC class II molecule, has been implicated in B-cell activation, as well as survival, with the chemokine MIF as the known ligand of CD74/CD44 complexes [1,3]. To investigate the effect of milatuzumab on the proliferation of CD19^+ ^B cells, CFSE-labeled PBMCs were stimulated *in vitro *with IL-2, IL-10, F(ab)_2_, and CpG, and cultured for 7 days with or without unconjugated milatuzumab and with IVIG as a control. The percentage of proliferating B cells was determined (Figure 2A); simultaneously, the concentration of endogenously produced MIF - a ligand of CD74 produced by the cultured PBMCs and potential confounding factor of this experimental setting - in the supernatants was quantified (Figure 2B).

Almost all CD19^+ ^B cells (98.1% ± 0.6%, mean ± SD) proliferated upon culturing PBMCs in the absence of milatuzumab or IVIG. In comparison with this baseline proliferation rate as well as with IVIG control (96.3% ± 1.2%), the presence of milatuzumab decreased the proportion of proliferating CFSE^reduced ^B cells modestly in all samples (93.7% ± 3.5%; *P *< 0.01). In contrast, neither milatuzumab nor IVIG had any influence on the concentration of MIF in the supernatants analyzed (Figure 2B). Thus, the effects observed on B-cell proliferation were not caused by modified MIF production in the respective cultures. Notably, the proportion of dead CD19^+ ^B cells, identified as high positive staining with DAPI, was not altered by milatuzumab (19.3% ± 3.8%) or IVIG (20.7% ± 4.3%) in comparison with control cells not exposed to either one (21.2% ± 5.0%) (Figure 2C). Thus, CD74 targeting by milatuzumab inhibits B-cell proliferation to a moderate, but higher, degree in comparison with controls.

### Anti-CD74 targeting changes the migration properties of CD19^+ ^B cells, especially of CD19^+^/CD27^- ^naïve B cells *in vitro*

The impact of CD74 targeting on B-cell *in vitro *migration and expression of adhesion molecules possibly changing B-cell trafficking also was evaluated. Transwell migration assays were used to determine the effect of milatuzumab on the migration of B cells toward the chemokine CXCL12 (ligand of CXCR4). Therefore, PBMCs were incubated with or without unconjugated milatuzumab or control IgG1/IVIG, and the migration of B cells through a fibronectin-coated transwell insert was assessed. Within these experiments, chemokine-independent baseline migration and CXCL12-dependent migration were quantified. Effective CXCL12-dependent migration of B cells was determined by subtracting individual baseline migration for each condition from the total migration toward CXCL12. The frequencies of migrated CD19^+ ^B cells and their CD27^- ^naïve and CD27^+ ^memory subpopulations are summarized in Figure 3A (baseline migration) and Figure 3B (net migration as the difference of actual minus baseline migration). Baseline migration of CD19^+ ^B cells (without CXCL12 in the lower chamber) was enhanced by milatuzumab from 2.7% ± 2.3% (mean ± SD) to 7.3% ± 3.4% (CD27^- ^B cells: 2.1% ± 1.8% to 7.0% ± 3.2%; CD27^+ ^B cells: 4.0% ± 3.8% to 8.0% ± 4.3%), which is consistent with recent observations of increased spontaneous chemokinesis of CD74^-/- ^macrophages [23], whereas IgG1 or IVIG did not influence baseline migration.

As noted above, CXCR4 has been reported as a co-receptor of CD74 and is the receptor of CXCL12 [15]. Therefore, subsequent studies addressed whether milatuzumab can impair CXCL12-dependent chemokinesis. When the net CXCL12-dependent migration was used, milatuzumab increased the frequency of migrated CD19^+ ^B cells significantly (*P *= 0.0078) from 15.8% ± 4.3% to 21.7% ± 6.6% (mean ± SD). In particular, the frequency of migrated CD27^- ^naïve B cells was substantially enhanced from 14.6% ± 5.4% to 22.9% ± 7.5% (*P *= 0.0078), whereas CD27^+ ^memory B cells showed no such increase in migration (18.2% ± 6.2% to 20.4% ± 6.7%; *P *= 0.1953). In other words, milatuzumab increased effective CXCL12-dependent migration of CD19^+ ^B cells 1.4-fold, of CD27^- ^naïve B cells 1.6-fold, and of CD27^+ ^memory B cells 1.1-fold. Since the expression of CD74 on CD27^+ ^memory was higher than on CD27^- ^naïve B cells, the capacity of the antibody to alter CXCL12-dependent migration appeared to be independent of or inversely proportional to the quantitative surface expression of CD74.

### Milatuzumab changes the surface expression of the adhesion molecules CD44, β7-integrin, and CD62L, especially on CD19^+^/CD27^- ^cells *in vitro*

To further delineate the effects of the anti-CD74 antibody on B cells in addition to moderately (but significantly) decreased proliferation and enhanced migration, its influence on the surface expression of several molecules was investigated. The MFIs and percentages of the co-receptors CD44, CXCR4, and CD9 on B cells, as well as the adhesion molecules β1-integrin, β7-integrin, and CD62L involved in lymphocyte trafficking, were determined after binding of the CD74 antibody.

Incubation with unconjugated milatuzumab altered the frequency of expression of the adhesion molecule CD44 on total CD19^+ ^B cells significantly, from 98.6% ± 0.6% to 97.4% ± 1.1% (mean ± SD; *P *= 0.0156) (Figure 4A). CD27^- ^naïve B cells, especially, expressed significantly less CD44 after incubation with milatuzumab (98.3% ± 0.7% to 96.9% ± 1.2%; *P *= 0.0156), whereas expression on CD27^+ ^memory B cells was unchanged (99.6% ± 0.5% to 99.5% ± 0.6%). However, analyses of respective MFIs showed that milatuzumab diminished the MFI of CD44 staining on CD19^+ ^B cells by an average of 10.7% (109.9 ± 20.1 to 98.0 ± 20.0) and the MFI of CD27^- ^naïve B cells by 12.1% (94.6 ± 15.2 to 83.2 ± 15.3), whereas the MFI of CD27^+ ^memory B cells showed only a modest decline of 5.3% (181.1 ± 34.0 to 172.2 ± 37.0). Overall, the expression level of CD74 and the reduction of CD44 expression after exposure to milatuzumab appeared to be independent or negatively correlated.

Additionally, the expression of β7-integrin and CD62L implicated in B-cell recirculation and tissue immigration was analyzed with regard to potential effects by milatuzumab. Whereas 71.1% ± 9.1% of CD19^+ ^B cells incubated without milatuzumab expressed β7-integrin (MFI of 73.2 ± 34.8), only 61.4% ± 8.3% of these cells expressed this adhesion molecule after milatuzumab incubation (*P *= 0.0078, MFI of 55.1 ± 22.6). The expression of β7-integrin on CD27^- ^naïve B cells was significantly reduced by milatuzumab (81.0% ± 8.7% compared with 71.3% ± 7.9%, *P *= 0.0391, MFI of 89.1 ± 37.2 compared with 63.7 ± 23.1) in contrast to CD27^+ ^memory B cells (49.8% ± 11.8% to 47.0% ± 9.0%, *P *= 0.1953, MFI of 53.6 ± 32.1 to 46.2 ± 23.4) (Figure 4B).

Furthermore, the expression of CD62L considered to be involved in systemic activation of B cells was diminished on CD19^+ ^B cells by milatuzumab (from 66.1% ± 11.1% to 53.2% ± 11.6%, *P *= 0.0039, MFI of 30.7 ± 13.0 to 16.9 ± 8.1), mainly by reduced expression on CD27^- ^B cells (69.6% ± 12.9% to 54.2% ± 12.9%, *P *= 0.0039, MFI of 33.2 ± 14.0 to 15.8 ± 8.3), but to a lesser extent on CD27^+ ^B cells (63.8% ± 14.6% to 57.0% ± 16.6%, *P *= 0.0547, MFI of 31.0 ± 19.4 to 22.9 ± 14.1) (Figure 4C).

Milatuzumab had no effect on the expression of CXCR4, β1-integrin, and CD9 in regard to either the percentage of expressing cells or the MFI values (data not shown). Here, β1-integrin and CD9 were expressed at higher levels on CD27^+ ^memory B cells and CXCR4 was expressed at lower levels in comparison with CD27^- ^naïve B cells, respectively. Incubation with IgG1 or IVIG also had no influence on B-cell surface expression or MFI values of any tested molecules. Additionally, the proportion of dead CD19^+ ^B cells, identified as high positive staining with DAPI, was not altered by milatuzumab or IgG1/IVIG in this experimental system.

## Discussion

This study identified, for the first time, a number of characteristics induced by targeting CD74 by using a humanized anti-CD74 antibody on human peripheral B-cell subsets. The data show a number of effects, including modest inhibition of proliferation, enhanced spontaneous migration, alterations of adhesion molecule expression, and CXCL12-dependent chemotaxis of B cells. Since it is well documented that CXCR4/CXCL12 is important for recruitment of lymphocytes, the present study suggests that treatment with milatuzumab might in fact deteriorate the recruitment of B cells to the sites of autoimmune inflammation or other involved organs or both.

Whereas binding of milatuzumab to human B cells and monocytes was detected, T cells did not show any binding. Notably, significantly enhanced migration toward CXCL12 and altered expression of the adhesion molecules, CD44, β7-integrin, and CD62L, were found, especially within CD19^+^/CD27^- ^naïve B cells but to a lesser extent on CD19^+^/CD27^+ ^memory B cells, which expressed CD74 at higher levels. Thus, there seems to be an inverse correlation between CD74 expression intensity and the effects observed after milatuzumab binding, suggesting that distinct intracellular pathways are induced in naïve versus memory B cells. Given the expression of CD74 on monocytes/macrophages and B cells, treatment with milatuzumab may permit combined targeting of the innate and adaptive immune systems and thus may be promising for treating autoimmunity, as exemplified by successful anti-cytokine treatment.

### Distinct surface expression of CD74 between CD19^+^/CD27^- ^naïve versus CD19^+^/CD27^+ ^memory B cells

CD74 expression was detected on monocytes and B cells (Figure 1A), consistent with previous reports [1], and entailed a study on its effects on B cells. The binding of milatuzumab to monocytes could be partially blocked with unconjugated milatuzumab as well as FcR-blocking reagents. However, the binding of milatuzumab on B cells was blocked completely and confirmed its specificity. Notably, it was identified that CD74 binding intensity was lower on CD27^- ^naïve versus CD27^+ ^memory cells. This observation led to the hypothesis that milatuzumab may have distinct effects on these B-cell subsets and hence they were analyzed separately. It was recently reported that CD74, together with CD44, is upregulated in the kidney and hippocampi of diseased lupus-prone mice and was reduced by treatment with the tolerogenic peptide hCDR1 [24]. These data, together with the present results, suggest that CD74 may be a suitable target for treating autoimmune diseases, but further *in vitro *data on monocytes are required.

### Milatuzumab inhibits the proliferation of CD19^+ ^B cells *in vitro *and changes the expression of CD44 on CD19^+^/CD27^- ^B cells

A potential function of CD74 is regulation of B-cell activation on the basis of its implications in MHC class II presentation. Therefore, the percentage of proliferating cells stimulated with CpG, IL-2, F(ab)_2_, and IL-10 in the presence of milatuzumab was studied. As a result, a modest decrease of proliferation induced by milatuzumab by using CFSE labeling could be detected. This is consistent with other studies applying this antibody and analyzing proliferation and survival of malignant B cells by using [^3^H]-thymidine uptake assays [2,4]. However, the use of CFSE in our experiments permitted a clearer quantification of proliferation. In addition, higher CD74 expression by B-cell malignancies does not allow a comparison with non-malignant cells having a lower baseline CD74 expression, although in both cases reduced proliferation could be observed after CD74 ligation. In this regard, another study using an activating anti-CD74 antibody showed enhanced proliferation of mouse splenocytes [7]. Recently, it was reported that the cytokine midkine and its receptor-type tyrosine phosphatase ζ (RPTPζ) are important regulators of B-cell survival. Since this phosphatase is part of an MIF/CD74-induced survival cascade [25], an interaction with CD74 ligation might be possible. The present study, however, suggests that targeting CD74 can partially inhibit proliferation of normal human B cells, but further studies, including the demonstration of its relevance and net effect *in vivo*, are necessary.

The surface expression of CD44 known as a co-receptor of CD74 has also been analyzed in CD27^- ^naïve and CD27^+ ^memory B-cell subsets. Notably, CD44 was expressed at higher levels on CD27^+ ^memory B cells than on CD27^- ^naïve B cells. Thus, higher CD74 and CD44 expressions mark CD27^+ ^memory B cells compared with CD27^- ^naïve B cells and differ from those of malignant lymphoma B cells overexpressing CD74 [1,26]. Milatuzumab decreased the expression of CD44, a molecule also known to be involved in homing to lymphoid tissues [27], on CD27^- ^naïve B cells but not on CD27^+ ^memory B cells (Figure 4A). This effect appeared to be a possible consequence of a distinct regulation due to the blocking of CD74. In this context, studies in CD74-deficient mice [28] showed that a greater number of activated dendritic cells migrated in the lymph nodes. Consistent with that, a decrease of PBMCs in the blood of cynomolgus monkeys treated with milatuzumab was reported [1]. All of these data are consistent with effects on the redistribution of immune cells under blockade of CD74 but require confirmation by detailed *in vivo *human studies.

An important aspect of the proliferation studies was to evaluate the extent to which the chemokine MIF, as a potential ligand of the receptor complex formed by CD74 and CD44, could have influenced the results of the proliferation studies. Because the MIF concentration was not changed after milatuzumab incubation in cultures over the course of 7 days, it appears unlikely that MIF production by co-cultured PBMCs was a confounding factor for any of the effects induced by milatuzumab on B cells. These data are very consistent with a recent report on macrophage function *in vivo *and *in vitro*; using CD74^-/- ^and MIF^-/- ^mice, the report demonstrated that MIF and CD74 mediate their effects via common and independent mechanisms [23].

### Milatuzumab changes migration and expression of adhesion molecules β7-integrin and CD62L mainly of CD19^+^/CD27^- ^naïve B cells

It has been reported that CD74 targeting can lead to changes in macrophage [23] as well as dendritic cell migration [28], and possibly changes of adhesion also may contribute to these effects. In the present study, analysis of the expression of CXCR4, a molecule known to be involved in the migration of B cells toward CXCL12 [16,17] and able to form a complex with CD74 [15], yielded a higher expression on CD27^- ^naïve B cells in comparison with CD27^+ ^memory B cells. This is consistent with previous data [17,29] and suggests different migration properties and effects on B-cell trafficking by targeting CD74. Indeed, milatuzumab enhanced migration of CD27^- ^naïve, but not those of CD27^+ ^memory, B cells without alteration of CXCR4 expression, indicating that its surface expression does not necessarily correlate with its migrational capacity [17,30-32]. These findings suggest that blocking of CD74 in the CD74-CXCR4 complexes increases the amount of 'functionally free' CXCR4 for migration or modulation of intracellular signaling pathways. In this context, it was recently suggested that MIF-mediated activation of the c-Jun N-terminal kinase (JNK) signaling pathway is dependent on CD74 and CXCR4 in T cells and fibroblasts. In CD74-deficient mouse embryonic fibroblasts, diminished JNK activation was observed after MIF stimulation. Pre-incubation with a neutralizing monoclonal anti-CXCR4 antibody completely blocked the upregulation of JNK phosphorylation after MIF exposure in Jurkat T cells [33]. Although such data are not available for B cells, it is conceivable that milatuzumab targeting CD74 could also interfere with this signaling pathway.

Of note, there was enhanced spontaneous migration after binding of milatuzumab in the absence of any chemokine, and this is very consistent with macrophage and dendritic cell data indicating that CD74 has antagonistic effects on cell migration. CD74-deficient dendritic cells [28] and CD74^-/- ^macrophages [23] have been shown to migrate faster than wild-type cells, whereas an activating anti-CD74 antibody has been reported to induce a dose-dependent inhibition of *in vitro *migration of mesenchymal stem cells [34]. The present study extends the role of CD74 in migration to B cells, whereas an inverse relationship also seems to be apparent. In this regard, the observed increase of migration of CD19^+ ^B cells by adding milatuzumab appears to be involved in adherence to high endothelial venules (HEVs) within mucosal tissue [35]. Therefore, milatuzumab seems to alter cell migration that is possibly dependent on the cell type and related to changes of intracellular signaling pathways. In this context, an enhanced migration does not necessarily imply increased migration into lymphoid organs provided that the extent to which this coincides with qualitative differences in migration is not clear.

The anti-CD74 antibody was also able to decrease the surface expression of β7-integrin as well as CD62L, mainly on CD27^- ^naïve B cells *in vitro*. β7-integrin, an adhesion molecule binding to MadCAM-1 in cooperation with α4-integrin, is known to be involved in adherence to HEVs within mucosal tissues [35]. Thus, the reduced expression induced by milatuzumab *in vivo *may impair cell migration and prevent their proper participation in immune responses. The changes of the expression of CD62L may change the attachment of leukocytes to HEVs of peripheral lymph nodes, mucosal lymph nodes, Peyer's patches, and the spleen [36]. Notably, expression of two fibronectin-binding molecules [37,38], β1-integrin and CD9, was not changed by milatuzumab (data not shown), so the changes observed for CD62L and β7-integrin were not the result of a non-specific downregulation of surface receptors but were likely under the control of intracellular signaling pathways. In this context, it was recently suggested that treatment with a tolerogenic peptide resulted in reduced CD74 and CD44 expression [24] and may lead, via NF-κB-dependent signaling, to reduced CD69 expression and, consequently, to reduced anti-double-stranded DNA autoantibody production in a lupus mouse model. Another study [7] found that intramembraneous cleavage after CD74/MIF binding leads to NF-κB activation via the Syk/Akt phosphorylation pathway. However, intracellular signaling downstream of CD74 appears to be very complex and may differ if MIF, CD44, or CXCR4 is involved. Therefore, the complex signaling pathways of CD74 in B cells require comprehensive studies that consider the context.

Since the effects of milatuzumab on adhesion molecule expression and migration were observed for CD27^- ^naïve B cells, it is likely that their impaired trafficking leads to the prevention of ongoing immune memory induction and thereby could lead to successful immune intervention. Further studies, including *in vivo *data that also take into account the complex interaction with other immune and non-immune cells expressing CD74 and targeted by milatuzumab, are needed to fully delineate the potential of anti-CD74 therapy in autoimmune diseases. Milatuzumab has a different mechanism of action than that of the B cell-depleting anti-CD20 antibody, rituximab. Thus, milatuzumab may be an alternative treatment of systemic lupus erythematosus or other autoimmune diseases, offering a co-targeting of monocytes/macrophages and B cells.

## Conclusions

Milatuzumab leads *in vitro *to modestly reduced proliferation, to alterations in migration, and to adhesion molecule expression preferentially of CD27^- ^naïve B cells. By targeting and consequently modifying functions of B cells and, potentially, monocytes/macrophages, it may change important migrational functions and thus may be a promising candidate antibody for treatment of autoimmune diseases.

## Abbreviations

BSA: bovine serum albumin; CFSE: carboxyfluorescein succinimidyl ester; CLL: chronic lymphocytic leukemia; CpG: cytosine-phosphatidyl-guanosine; DAPI: 4,6 diamidino-2-phenylindole; F(ab)_2_: protein of two antigen-binding fragments; FITC: fluorescein isothiocyanate; HEV: high endothelial venule; IL: interleukin; IVIG: intravenous immunoglobulin; JNK: c-Jun N-terminal kinase; MFI: (geometric) mean fluorescence intensity; MHC: major histocompatibility complex; MIF: macrophage migration inhibitory factor; NF-κB: nuclear factor-kappa-B; PBMC: peripheral blood mononuclear cell; PBS: phosphate-buffered saline; PE: phycoerythrin; SD: standard deviation.

## Competing interests

TD has served as principal investigator of a systemic lupus erythematosus study that investigated epratuzumab and that was funded by Immunomedics, Inc. DMG has a management role and owns stock in Immunomedics, Inc. All other authors declare that they have no competing interests.

## Authors' contributions

DF and DB carried out the experiments, performed statistical analyses, discussed results, made the figures, and prepared the manuscript. KR and CG participated in part of the experiments (transwell migration assays, fluorescence-activated cell sorting, and incubation experiments) and provided technical support. CD carried out the determination of MIF concentration. HEM, GRB, AS, and DMG discussed results and participated in the design of the study. TD conceived of the study and participated in its design and coordination, discussed results, and was responsible for supervision and manuscript preparation. All authors read and approved the final manuscript.
